# Characteristics and outcome‐related factors of seizure at the first onset of autoimmune encephalitis: A retrospective study

**DOI:** 10.1111/cns.13633

**Published:** 2021-03-08

**Authors:** Yilin Wang, Xin Li, Pingping He, Jiangning Yin, Ruofei Dong, Yu Fu, Hong Zhang

**Affiliations:** ^1^ Department of Neurology Shengjing Hospital of China Medical University Shenyang China

**Keywords:** antiepileptic drugs, autoimmune encephalitis, immunotherapy, seizure outcome, status epilepticus

## Abstract

**Aims:**

Seizure outcome of autoimmune encephalitis (AE) varies from seizure‐free to refractory epilepsy, and the associated factors remain unclear. We aimed to describe seizure characteristics, identify seizure outcome‐related factors, and discuss the medication strategy of antiepileptic drugs (AEDs) at the first onset of AE.

**Methods:**

We retrospectively studied the data of 86 patients with clinically diagnosed AE. The clinical characteristics were described using a chi‐square test. Seizure outcome‐related factors were assessed using multivariable logistic regression analysis.

**Results:**

56 patients were finally enrolled, with antibodies to N‐methyl‐D‐aspartate receptor found in 29, to γ‐aminobutyric acid receptor B found in 13, and to leucine‐rich glioma‐inactivated protein 1 found in 14. Status epilepticus occurrence and onset with seizure lead to a poor seizure outcome, while administration of human gamma globulin and a low antibody titer contributed to a good seizure outcome.

**Conclusions:**

In the acute phase, seizure characteristics may be considered in the utilization of AEDs. For patients with seizure‐free status in the acute phase, clinical manifestation (onset with seizure or not, whether status epilepticus occurs or not), therapy regimen (human gamma globulin administered or not), and antibody titer may be considered when formulating the strategy for withdrawal of AEDs post‐acute phase.

## Graphical abstract

In the acute phase, seizure characteristics may be considered in the utilization of antiepileptic drugs. For patients who have achieved seizure‐free status in the acute phase of autoimmune encephalitis, onset with seizure or not, status epilepticus occurs or not, human gamma globulin administered or not and antibody titer may be considered in the withdrawal strategy of AEDs after the acute phase.

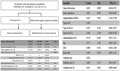

## 1 INTRODUCTION

In recent years, autoimmune encephalitis (AE) has attracted attention as a new and rarely described group of neurological inflammatory diseases associated with specific autoantibodies^1^ and multiple co‐infections.^2^ Common clinical manifestations include seizures, movement disorders, abnormal behavior, speech dysfunction, and autonomic dysfunction.^3^ With the development of biochemical assays, AE can be divided into many types,^4^ and anti‐N‐methyl‐D‐aspartate receptor (NMDAR), anti‐γ‐aminobutyric acid B receptor (GABABR), and anti‐leucine‐rich glioma‐inactivated protein 1 (LGI1) are the most common.^4^ The acute phase of AE is defined as the first 3 months after the onset of symptoms. In this phase, seizure is frequently observed and may be the initial symptom.^4^ In the more serious cases, patients may experience status epilepticus (SE).^5^ After the acute phase, some patients may achieve seizure‐free status with or without antiepileptic treatment, whereas others may progress to refractory epilepsy despite long‐term antiepileptic treatment. Given that the characteristics and seizure outcomes vary by type of AE and patient,^6^ individualized treatment is necessary. Some studies speculate that most patients with AE do not require long‐term use of antiepileptic drugs (AEDs),^7^, ^8^, ^9^ particularly for patients who have achieved seizure‐free status in the acute phase. Currently, in the absence of clear guidelines, the treatment duration and selection of AEDs have been controversial. In this study, we aimed to describe seizure characteristics, identify factors affecting outcomes related to seizures and discuss the therapeutic strategy of AEDs at the first onset of AE.

## 2 METHODS

### 2.1 Subjects

The Ethics Committee of the Shengjing Hospital of China Medical University granted ethical approval for this retrospective study (2020PS519K) and waived the requirement for written informed consent. The study was performed in accordance with the Declaration of Helsinki. We retrospectively reviewed the records of patients with AE who were treated at our hospital between January 2013 and January 2019. The patients included in this study met the diagnostic criteria for AE published in 2016.^10^ The exclusion criteria were as follows: (1) no seizure on record; (2) a history of AE or epilepsy; (3) a history of other diseases that can cause seizure, such as stroke, cerebrovascular malformation, and brain trauma; (4) types of AE that were not anti‐NMDAR, anti‐GABABR, or anti‐LGI1 encephalitis; (5) tumor; (6) incomplete clinical data; or (7) lost to follow‐up.

### 2.2 Clinical data collection

The following basic clinical data were collected: sex, age at onset, clinical symptoms, imaging findings, laboratory tests, cerebrospinal fluid (CSF) examination, tests for autoantibodies in the CSF, treatment regimen, and prognosis. Medical information was collected from electronic databases or by telephone interview with patients and relatives.

### 2.3 Definitions

Onset symptoms were divided into “with seizure” and “without seizure” according to whether the patients began having seizures or not. Laboratory tests and CSF examination were divided into abnormal and normal based on reference intervals. Imaging findings were divided into abnormal and normal based on the results. Clinical symptoms were coded as “with” or “without” according to the presence or absence of symptoms. With regard to treatment regimen, the administration of human gamma globulin or immunosuppressive agents were coded as “with” or “without” according to use or non‐use. AED regimens were divided into early withdrawal (EW) and late withdrawal based on whether withdrawal occurred within 3 months of onset. Scores on the Modified Rankin Scale were measured at discharge. Antibody scoring was defined according to the titer. The titer in CSF≤1:10 was scored as (+); >1:10 and ≤1:100, (++); and >1:100, (+ + +).

### 2.4 Outcome assessment

An outcome was assessed based on whether the patient achieved seizure‐free status after EW or not. Achievement of seizure‐free status after EW was defined as AEDs withdrawn within 3 months and absence of seizure attack for at least 1 year after the last use of AEDs. Other outcomes, such as seizure attack after EW, seizure‐free with utilization of AEDs for more than 3 months, and seizure attack with utilization of AEDs for more than 3 months, were defined as failure to achieve seizure‐free status after EW.

### 2.5 Statistical analysis

Continuous variables were defined as means ± standard deviations; categorical variables were described as counts and percentages. A chi‐square test was performed to describe the seizure characteristics observed in AE. A multivariate logistic regression analysis was used to assess the independent predictors of seizure outcome, and the results are provided as odds ratios with 95% confidence intervals. Statistical analyses were performed using SPSS Statistics 23.0 software (IBM Corp.). *p* < 0.05 was considered statistically significant.

## 3 RESULTS

### 3.1 Baseline characteristics of different types of AE

**FIGURE 1:**
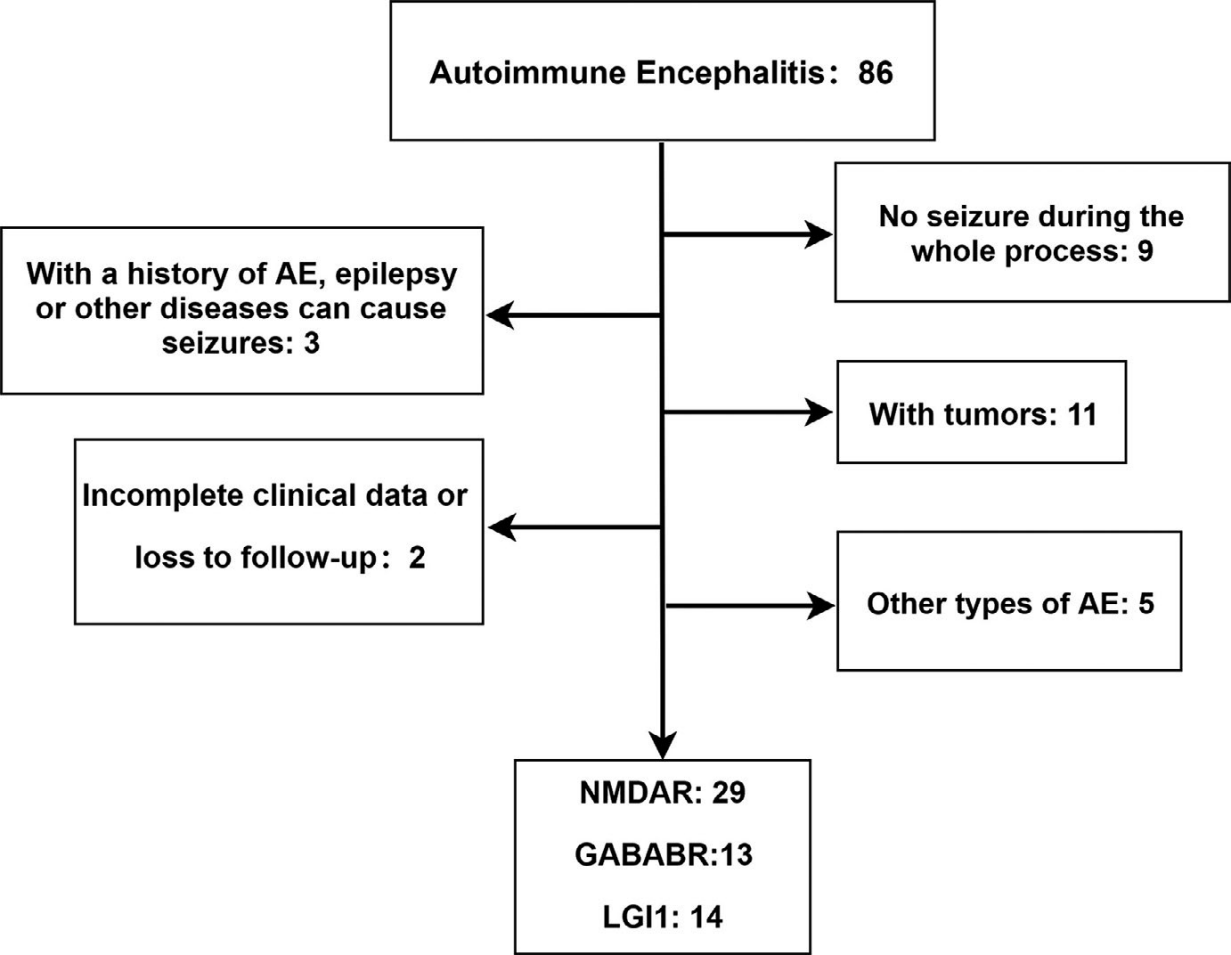
Flow diagram of patient inclusion and grouping. AE, autoimmune encephalitis; GABABR, γ‐aminobutyric acid B receptor; LGI1, leucine‐rich glioma‐inactivated protein 1; NMDAR, N‐methyl‐D‐aspartate receptor

A total of 86 patients with confirmed AE were identified in our hospital records between January 2013 and January 2019. According to the exclusion criteria, 30 patients were excluded. In total, 56 patients were finally included (Figure 1). Twenty‐nine patients had anti‐NMDAR encephalitis (16 female and 13 male), 13 had anti‐GABABR encephalitis (4 female and 9 male), and 14 had anti‐LGI1 encephalitis (7 female and 7 male). The demographic features of these patients are summarized in Table 1. Antibody titer and blood sodium level differed significantly among 3 groups .

**TABLE 1:** Patient baseline characteristics by type of autoimmune encephalitis (AE)

Baseline characteristic	NMDAR (*n* = 29)	GABABR (*n* = 13)	LGI1 (*n* = 14)	*p* value
Sex: Male, No. (%)	13 (44.8)	9 (69.2)	7 (50.0)	0.361
Age, No. (%)				
0–14	14 (48.3)	4 (30.8)	2 (14.3)	0.084
>14	15 (51.7)	9 (69.2)	12 (85.7)	
Movement disorder, No. (%)	8 (27.6)	3 (23.1)	3 (21.4)	0.923
Dysarthria, No. (%)	9 (31.0)	3 (23.1)	6 (42.9)	0.521
Mood disorders, No. (%)	22 (75.9)	6 (46.2)	11 (78.6)	0.118
Fever (>37.5℃), No. (%)	15 (51.7)	7 (53.8)	5 (35.7)	0.553
Laboratory tests (abnormal), No. (%)				
Blood potassium level	2 (6.9)	2 (15.4)	3 (21.4)	0.424
Blood sodium level	3 (10.3)	2 (15.4)	6 (42.9)	0.040
Blood chlorine level	7 (24.1)	2 (15.4)	6 (42.9)	0.291
CSF tests (abnormal), No. (%)				
Pressure	17 (58.6)	7 (53.8)	5 (35.7)	0.365
WBC count	16 (55.2)	9 (69.2)	6 (42.9)	0.433
Protein level	4 (13.8)	6 (46.2)	3 (21.4)	0.074
Glucose level	3 (10.3)	0	1 (7.1)	0.799
Chloride level	2 (6.9)	1 (7.7)	3 (21.4)	0.456
Antibody titer, No. (%)				
+	18 (62.1)	3 (23.1)	3 (21.4)	0.024
+ +	6 (20.7)	7 (53.8)	9 (64.3)	
+ + +	5 (17.2)	3 (23.1)	2 (14.3)	
Administration of HGG, No. (%)	11 (37.9)	5 (38.5)	7 (50.0)	0.735
Administration of IA, No. (%)	11 (37.9)	4 (30.8)	2 (14.3)	0.278
Antiepileptic drugs, No. (%)				
Early withdrawal (≤3 months)	16 (55.2)	5 (38.5)	8 (57.1)	0.544
Late withdrawal (>3 months)	13 (44.8)	8 (61.5)	6 (42.9)	
Modified Rankin Scale, No. (%)				
0–2	24 (82.8)	11 (84.6)	12 (85.7)	1.000
3–6	5 (17.2)	2 (15.4)	2 (14.3)	

Abbreviations: CSF, cerebrospinal fluid; GABABR, γ‐aminobutyric acid B receptor; HGG, human gamma globulin; IA, immunosuppressive agent; LGI1, leucine‐rich glioma‐inactivated protein 1; NMDAR, N‐methyl‐D‐aspartate receptor; WBC, white blood cell.

### 3.2 Seizure characteristics of different types of AE

Seizure characteristics are presented in Table 2. Compared with the other two groups, the patients in the LGI1 group were more prone to onset with seizure (71.4%, *n* = 14), and the patients in the GABABR group were more prone to SE occurrence (46.2%, *n* = 13). Faciobrachial dystonic seizures (FBDS) were only observed in the LGI1 group (*p* < 0.0001). The seizure type in the GABABR group was generalized. Focal seizures were more common in the NMDAR group (48.3%, *n* = 29). Abnormal electroencephalogram (EEG) results (57.1%, *n* = 14) and brain magnetic resonance imaging (MRI) results (71.4%, *n* = 14) were both more common in the LGI1group than in the other groups. The patients in the GABABR group had a relatively low frequency of achieving seizure‐free status with EW (30.8%, *n* = 13) compared with the other groups.

**TABLE 2:** Seizure characteristics by type of AE

Seizure characteristic	NMDAR (*n* = 29)	GABABR (*n* = 13)	LGI1 (*n* = 14)	*p* value
Onset with seizure, No. (%)	12 (41.4)	7 (53.8)	10 (71.4)	0.176
Status epilepticus, No. (%)	11 (37.9)	6 (46.2)	4 (28.6)	0.665
Main seizure type, No. (%)				
FBDS, No. (%)	0	0	6 (42.9)	<0.0001
Generalized seizure, No. (%)	15 (51.7)	13 (100.0)	5 (35.7)	
Focal seizure, No. (%)	14 (48.3)	0	3 (21.4)	
EEG (abnormal), No. (%)	14 (48.3)	6 (46.2)	8 (57.1)	0.820
Brain MRI (abnormal), No. (%)	17 (58.6)	6 (46.2)	10 (71.4)	0.424
Seizure‐free with EW, No. (%)	15 (51.7)	4 (30.8)	8 (57.1)	0.338

Abbreviations: AE, autoimmune encephalitis; EEG, electroencephalogram; EW, early withdrawal; FBDS, faciobrachial dystonic seizure; GABABR, γ‐aminobutyric acid B receptor; LGI1, leucine‐rich glioma‐inactivated protein 1; MRI, magnetic resonance imaging; NMDAR, N‐methyl‐D‐aspartate receptor.

### 3.3 Seizure outcome‐related factors in AE

A univariate analysis was performed for preliminary identification of factors related to seizure outcome. Onset symptom, SE, human gamma globulin administration, and antibody titer were identified at a significance level of *p* < 0.2 (Table 3). According to previous studies, age at onset,^4^ use of immunosuppressive agents,^9^ EEG results,^11^ brain MRI results,^11^ and type of AE^11^ are related to seizure outcome. Accordingly, these variables were retained for the multivariable logistic regression analysis, despite *p* values >0.2. As shown in Table 4, at a significance level of *p* ≤ 0.05, onset with seizure, SE, human gamma globulin use, and antibody titer were related to seizure outcome in AE. The odds of patients with onset with non‐seizure symptoms achieving seizure‐free status after EW were 1:0.099, 10.10 times greater than those of patients with onset with seizure. The odds of patients without SE achieving seizure‐free status after EW were 1:0.091, 10.99 times greater than those of patients with SE. The odds of patients treated with human gamma globulin achieving seizure‐free status after EW were 5.852 times greater than those of patients not treated with these. Antibody titer was also associated with seizure outcome. The lower the concentration, the more likely it was for patients to achieve seizure‐free status after EW. The odds of patients with an antibody titer of (+) achieving seizure‐free status after EW were 1:0.139, 7.19 times greater than those of patients with a (++) titer and 1:0.033, or 30.30 times greater than those of patients with a (+++) titer.

**TABLE 3:** Univariate analysis of outcome‐related factors of seizures

Variables	Seizure‐free after EW		*n*	*p* value
	No (*n* = 29)	Yes (*n* = 27)		
Onset symptom, No. (%)				
Not seizure	11 (40.7)	16 (59.3)	27	0.113
Seizure	18 (62.1)	11 (37.9)	29	
SE, No. (%)				
Without SE	15 (42.9)	20 (57.1)	35	0.088
With SE	14 (66.7)	7 (33.3)	21	
HGG, No. (%)				
Without HGG	21 (63.6)	12 (36.4)	33	0.036
With HGG	8 (34.8)	15 (65.2)	23	
Type of AE, No. (%)				
NMDAR	14 (48.3)	15 (51.7)	29	0.351
GABABR	9 (69.2)	4 (30.8)	13	
LGI1	6 (42.9)	8 (57.1)	14	
IA, No. (%)				
Without IA	19 (48.7)	20 (51.3)	39	0.488
With IA	10 (58.8)	7 (41.2)	17	
Sex, No. (%)				
Male	14 (48.3)	15 (51.7)	29	0.586
Female	15 (55.6)	12 (44.4)	27	
Age (years, mean ±SD)	33.5 ± 23.9	35.1 ± 27.9		0.811
Seizure type, No. (%)				
FBDS	1 (16.7)	5 (83.3)	6	0.250
Generalized seizure	19 (57.6)	14 (42.4)	33	
Focal seizure	9 (52.9)	8 (47.1)	17	
MRS, No. (%)				
0–2	20 (42.6)	27 (57.4)	47	0.999
3–6	9 (100)	0	9	
Movement disorder, No. (%)				
Without	23 (54.8)	19 (45.2)	42	0.442
With	6 (42.9)	8 (57.1)	14	
Fever (>37.5℃), No. (%)				
Without	16 (55.2)	13 (44.8)	29	0.599
With	13 (48.1)	14 (51.9)	27	
EEG, No. (%)				
Normal	15 (53.6)	13 (46.4)	28	0.789
Abnormal	14 (50.0)	14 (50.0)	28	
Brain MRI, No. (%)				
Normal	10 (43.5)	13 (56.5)	23	0.301
Abnormal	19 (57.6)	14 (42.4)	33	
Dysarthria, No. (%)				
Without	20 (52.6)	18 (47.4)	38	0.854
With	9 (50.0)	9 (50.0)	18	
Blood potassium level, No. (%)				
Normal	25 (51.0)	24 (49.0)	49	0.762
Abnormal	4 (57.1)	3 (42.9)	7	
Blood sodium level, No. (%)				
Normal	25 (55.6)	20 (44.4)	45	0.260
Abnormal	4 (36.4)	7 (63.6)	11	
Blood chlorine level, No. (%)				
Normal	23 (56.1)	18 (43.9)	41	0.289
Abnormal	6 (40.0)	9 (60.0)	15	
CSF pressure, No. (%)				
Normal	12 (44.4)	15 (55.6)	27	0.290
Abnormal	17 (58.6)	12 (41.4)	29	
CSF WBC count, No. (%)				
Normal	11 (44.0)	14 (56.0)	25	0.297
Abnormal	18 (58.1)	13 (41.9)	31	
CSF protein level, No. (%)				
Normal	22 (51.2)	21 (48.8)	43	0.865
Abnormal	7 (53.8)	6 (46.2)	13	
CSF glucose level, No. (%)				
Normal	27 (51.9)	25 (48.1)	52	0.941
Abnormal	2 (50.0)	2 (50.0)	4	
CSF chloride level, No. (%)				
Normal	26 (52.0)	24 (48.0)	50	0.926
Abnormal	3 (50.0)	3 (50.0)	6	
Antibody titer, No. (%)				
+	8 (33.3)	16 (66.7)	24	0.061
+ +	14 (63.6)	8 (36.4)	22	
+ + +	7 (70.0)	3 (30.0)	10	

Abbreviations: AE, autoimmune encephalitis; CSF, cerebrospinal fluid; EEG, electroencephalogram; EW, early withdrawal; FBDS, faciobrachial dystonic seizure; GABABR, γ‐aminobutyric acid B receptor; HGG, human gamma globulin; IA, immunosuppressive agent; LGI1, leucine‐rich glioma‐inactivated protein 1; MRI, magnetic resonance imaging; MRS, modified Rankin scale; NMDAR, N‐methyl‐D‐aspartate receptor; SE, status epilepticus; WBC, white blood cell.

**TABLE 4:** Multivariable logistic regression results for seizure outcome‐related factors

Variable	*p* value	OR	95% CI
Onset with seizure	0.023	0.099	0.014–0.723
Status epilepticus	0.031	0.091	0.010–0.807
Administration of HGG	0.027	5.852	1.224–27.983
Type of AE	0.290		
Type of AE (1)	0.347	0.356	0.041–3.075
Type of AE (2)	0.442	2.339	0.268–20.387
Administration of IA	0.115	0.229	0.037–1.431
Age	0.272	1.022	0.983–1.061
EEG	0.080	0.143	0.016–1.260
Brain MRI	0.305	0.439	0.091–2.121
Antibody titer	0.022		
Antibody titer (1)	0.042	0.139	0.021–0.927
Antibody titer (2)	0.011	0.033	0.002–0.460

Abbreviations: AE, autoimmune encephalitis; CI, confidence interval; EEG, electroencephalogram; HGG, human gamma globulin; IA, immunosuppressive agent; MRI, magnetic resonance imaging; OR, odds ratio.

## 4 DISCUSSION

In this retrospective study, by collecting seizure‐related details of patients, we were able to describe the seizure‐related characteristics of the three main types of AE. We observed similarities and differences among three types of AE. Moreover, by multivariable logistic regression analysis, we identified several factors associated with seizure outcome: SE occurrence and onset with seizure may lead to a poor seizure outcome, while human gamma globulin administration and a lower antibody titer may contribute to a good seizure outcome. Combined with our other results, these findings suggest that seizure characteristics may be considered in seizure management, and outcome‐related factors may be considered when formulating an AED withdrawal strategy.

Given the small sample size, we used a descriptive, not statistical, analysis combined with a literature search to identify the possible characteristics of seizures related to the three main types of AE. We mainly focused on the onset symptoms, seizure type, EEG characteristics and MRI results. The onset symptoms are shown in Table 2. The proportions of patients with onset with seizure in the three groups were 41.4% (NMDAR, *n* = 29), 53.8% (GABABR, *n* = 13), and 71.4% (LGI1, *n* = 14). According to the literature, anti‐NMDAR encephalitis usually begins with psychiatric symptoms and not seizure,^12^ while anti‐GABABR^13^ and anti‐LGI1 encephalitis^14^ usually begin with seizure. These trends can be observed in our results, despite the tendencies being slight in the NMDAR and GABABR groups. Regarding seizure type, our results show that in the group with anti‐NMDAR encephalitis, generalized seizures were the most common (51.7%, *n* = 29). Our findings are consistent with literature reports that generalized seizure,^15^ especially of the tonic‐clonic type, is commonly encountered.^16^ In the group with anti‐GABABR encephalitis, SE was more frequently observed (46.2%, *n* = 13) than in the other two groups. According to the literature, about 81% of these patients develop SE,^17^ which even caused mortality in some cases.^18^ In the group with anti‐LGI1 encephalitis, FBDS (42.9%, *n* = 14) was the most commonly observed seizure type and was only observed in this group. Consistently, the literature points out that FBDS, known as a unique feature of anti‐LGI1 encephalitis,^19^ is more frequently encountered than other seizure types.^20^ With respect to EEG data, our study found no group‐specific characteristics (see Table 2). According to the literature, however, focal slowwaves^21^ or generalized slowing^22^ are common in anti‐NMDAR encephalitis. Extreme delta brush, a known feature of anti‐NMDAR encephalitis,^23^ indicates a pooroutcome,^23^ despite being rarely detected.^24^ However, no classical extreme delta brush was detected in our study. In anti‐GABABR encephalitis, slow waves with a relatively wide range^25^ that are related to the severity of the disease are usually observed.^26^ While anti‐LGI1 encephalitis is characterized by slow wave activity from multifocal discharges,^27^ the main targets are the hippocampus^27^ and temporal regions.^28^ Interestingly, almost all the patients with FBDS present normal EEG recordings,^29^ which was also verified in our study. Abnormal MRI results, particularly in the hippocampus and temporal lobe, can be observed in all three types of AE,^16^, ^17^, ^30^, ^31^ which was also verified in our study. Moreover, hippocampal sclerosis due to seizure is commonly observed in both anti‐GABABR ^26^, ^32^ and anti‐LGI1 encephalitis in long‐term follow‐ups.^33^ In our study, no hippocampal sclerosis was detected, which may be attributed to the small sample size and short follow‐up period.

Our study suggests that onset with seizure and SE occurrence lead to a poor seizure outcome, while administration of human gamma globulin and a lower antibody titer may contribute to a good seizure outcome. Consistently, the literature suggest that SE is associated with long‐term chronic seizures^4^ and that autoimmune SE is refractory to AEDs.^34^ Moreover, timely termination of SE contributes to a good seizure outcome.^4^ In our study, human gamma globulin administration contributed to a good seizure outcome, whereas immunosuppressive agents did not. The literature indicates that immunotherapy is associated with a relatively good seizure outcome,^9^ but does not distinguish between the roles of human gamma globulin and immunosuppressive agents.

We also discovered that antibody titer is associated with seizure outcome. The lower the concentration, the more likely it was for patients to achieve seizure‐free status after EW. Consistently, the literature suggests that high antibody titer leads to poor outcomes.^35^, ^36^ However, some researchers question this relationship, given the persistence of autoantibodies long after remission.^37^, ^38^ We found that onset with seizure was negatively related to seizure outcome. We found no literature to date mentioning this result, and further studies are warranted.

In the present study, the results on analysis of MRI abnormalities, age, EEG, and AE type as seizure outcome‐related factors were unremarkable, which is inconsistent with previous literature. According to the literature, cortical abnormalities on MRI,^4^ older age,^4^ abnormal EEG findings, and the presence of anti‐GABABR antibody^11^ are significantly related to long‐term chronic seizures. The inconsistency of our MRI and EEG findings with those of previous studies may be attributed to a difference in the timepoint of the examination. In the early stage of AE, brain MRI and EEG results are usually normal and gradually present abnormalities with the progression of the disease. Moreover, different onset symptoms may be responsible for different timepoints of initial examination. For example, some patients may have an onset with fever or other symptoms mimicking the common flu, thereby delaying the first hospital visit and contributing to a more complete presentation of abnormalities on MRI and EEG. In contrast, patients with seizure onset visit the hospital in a timely manner and may present unremarkable MRI and EEG results at this early stage. Moreover, our divergent results on the influence of age and AE type may be explainable by the small sample size.

In clinical practice, we often prioritize AEDs with a relatively fast drug titration to control the seizures as soon as possible. Moreover, seizure type should also be considered during AED selection. For example, anti‐NMDAR encephalitis usually begins without seizure,^12^ and generalized seizure is commonly encountered subsequently.^15^ The timepoint and seizure type may be considered in AED utilization. Patients with anti‐GABABR encephalitis are vulnerable to SE^39^ and refractory seizures.^18^ For SE, timely immunotherapy is more effective than other therapies such as general anesthesia.^40^ Intravenous administration and rapid drug titration may be helpful as necessary.^41^ For anti‐LGI1 antibody encephalitis, hyponatremia is common,^6^ and some AEDs, such as carbamazepine and oxcarbazepine, may aggravate this condition.^42^ Additionally, severe cutaneous side effects^43^ are common with some AEDs. Our study suggests that, given the importance of human gamma globulin treatment, AEDs alone cannot contribute to a good seizure outcome in the acute phase. Additionally, the literature states that immunotherapy is time‐sensitive^7^ and either AEDs^44^ or immunotherapy alone^24^ is not recommended. Therefore, a timely combination of immunotherapy as the basic treatment and AEDs as an add‐on treatment is optimal.^45^ However, a previous study found that some AEDs with sodium‐channel blocking properties when administered alone have effectively controlled seizures in a few cases.^46^ Therefore, further studies to clarify the role of AEDs in AE treatment are needed.

After the acute phase, AED discontinuation may be considered in patients achieving seizure‐free status in the acute phase. Considering the factors related to seizure outcome identified in our study, we suggest discontinuation should be prioritized for patients with onset without seizure, without SE occurrence, with human gamma globulin administration and with low antibody titer. Additionally, another study has speculated that AE patients without epileptiform discharges on EEG or signs of inflammation on brain MRI are qualified for AED discontinuation.^19^ However, a larger‐sample replication was needed for verification of these views.

This study has several limitations. First, all 56 patients were enrolled at a single center, and a regional selection bias may therefore exist, especially since the sample size was small. Second, we did not consider the effect of hormones on seizure outcome, given that all 56 patients enrolled in our study had been treated with hormones. Third, few patients with other types of antibody, such as to contact protein‐like protein‐2, anti‐gamma‐aminobutyric acid A receptor, and anti‐alpha‐amino‐3‐hydroxy‐5‐methyl‐4‐isoxazolepropionic acid, were excluded, which may limit interpretation of the multivariate logistic regression result of the types of AE. Fourth, some seizure outcomes were evaluated based on telephone follow‐up; therefore, non‐motor seizures may have been overlooked. Finally, we did not assess the effect of tumor on seizure outcome. Given the inconsistent follow‐up time, some patients had completed surgery to remove tumors, whereas others had not. In this context, surgery may bias the effect of tumor on seizure outcome. Future studies should give more attention to the rare types of AE and to the effects of hormones and tumors.

## 5 CONCLUSIONS

In the acute phase of AE, seizure characteristics may be considered in the selection and utilization of AEDs. Onset with seizure and SE occurrence may lead to a poor seizure outcome, while human gamma globulin administration and low antibody titer may contribute to a good seizure outcome. For patients who have achieved seizure‐free status in the acute phase, the factors mentioned above may be considered in the withdrawal strategy of AEDs after the acute phase. Early identification of patients qualified to discontinue AEDs can avoid the additional adverse effects and high costs of AEDs, thereby easing the treatment burden borne by patients.

## CONFLICT OF INTEREST

The authors declare that there are no conflicts of interest.

## AUTHOR CONTRIBUTIONS

YL Wang collected the data, reviewed the literature, and drafted the manuscript; PP He, JN Yin, RF Dong, X Li, and Y Fu collected the data; H Zhang revised the manuscript.

## Data Availability

The data that support the findings of this study are available from the corresponding author upon reasonable request.
